# Identification of a systemic lupus erythematosus disease signature in resting naive B cells

**DOI:** 10.1172/JCI199528

**Published:** 2026-10-15

**Authors:** James R. Rose, Yusho Ishii, Arezou Khosroshahi, Sakeenah Hicks, Scott A. Jenks, Christopher D. Scharer, Ignacio Sanz, Jeremy M. Boss

**Affiliations:** 1 Department of Microbiology and Immunology,; 2 Division of Rheumatology, Department of Medicine, and; 3 Lowance Center for Human Immunology, School of Medicine, Emory University, Atlanta, Georgia, USA.

**Keywords:** Autoimmunity, Immunology, Adaptive immunity, B cells, Lupus

## Abstract

Systemic lupus erythematosus (SLE) is a chronic autoimmune condition with alternating phases of heightened symptoms and periods of disease inactivity. Our investigation focuses on the distinct molecular profiles of resting naive B cells from both active and inactive SLE states in female adults, primarily of Black ancestry. We identified a distinct transcriptome and epigenome during inactive SLE. Transcriptional patterns indicated an increase in distinct cytokine signaling pathway expression in SLE B cells, encompassing both common and disease activity–specific components. Specifically, while both disease states exhibited interferon signatures, increased expression of IL-12, TNF-α/NF-κB, and CD40 signaling pathway genes was enriched in inactive disease. While some regions exhibited comparable epigenetic features in both disease states, such as TLR7 signaling genes, others were distinct. Chromatin accessibility in inactive disease indicated the involvement of MEF2 and bHLH family factors, such as E2A/E2-2, while active disease regions were defined by IRF family factors. This study illuminates both disease-associated programs and molecular changes to cell activation pathways capable of directing B cells toward diverse effector fates in active or inactive SLE. Our data also offer insights of particular importance for the design and monitoring of B cell–targeting treatments.

## Graphical abstract

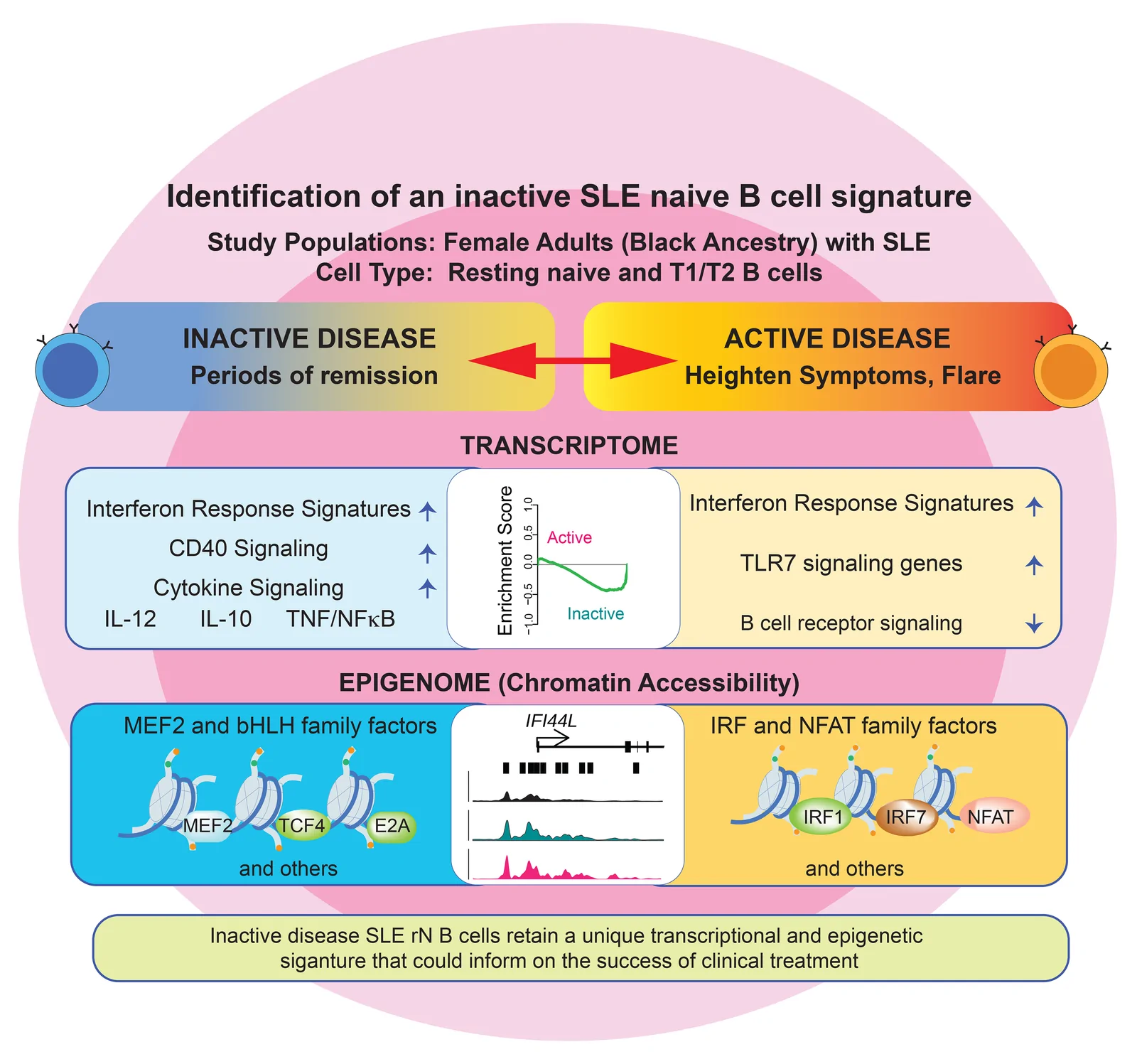

## Introduction

Systemic lupus erythematosus (SLE) is a heterogeneous autoimmune disease characterized by autoreactive antibodies produced by aberrant B cells. Its autoinflammatory symptoms are cyclical, alternating between high disease activity followed by periods of remission or low disease activity (1). In SLE, the proportion and quality of peripheral blood B cell populations are altered by both disease state and activity (2, 3). Notably, activated naive (aN) B cells (CD19^+^CD27^–^IgD^+^CD11c^+^TBET^+^CXCR5^–^) and their progeny, double-negative 2 (DN2) B cells (CD19^+^CD27^–^IgD^–^CD11c^+^TBET^+^CXCR5^–^) generated through extrafollicular immune reactions, are expanded in proportion to disease activity (2, 3). These B cell subsets have been shown to be poised to differentiate into plasmablasts in response to TLR7 signaling, a major SLE trigger, which also includes a strong genetic association with disease (4). Our earlier studies revealed the presence of a distinct transcriptome and epigenome in resting naive (rN) B cells in individuals with highly active SLE, in addition to a very similar transcriptome between SLE aN and DN2 cells (2, 5, 6). While these data showed an abnormal and epigenetically imprinted regulatory program in SLE naive B cells, the absence of inactive SLE in the study precluded a determination of whether the observed programs were induced by the disease state or were instead induced in response to specific stimulation during active disease (5, 6). Our early work also did not interrogate newly emerging transitional T1 and T2 B cells, which remain to be investigated in SLE. In a healthy immune system, rN B cells represent a preantigenic, unstimulated population. Accordingly, we postulated that differences in the regulatory programs of rN B cells in individuals with active and inactive/low SLE disease would reflect either cell-intrinsic disease-associated abnormalities or possibly differences in the cytokine milieu responsible for cytokine priming in subsequent B cell activation events during disease flares.

Several recent studies have centered on transcriptional regulation of immune cells from Japanese individuals with SLE, including elegant studies of sorted predefined populations, which uncovered both disease state and disease activity signatures (7, 8). Broadly defined, disease state is whether someone has SLE or not, whereas disease activity is a measurement of severity of a disease. Combined, these studies have identified different disease signatures in multiple cell types, with some differences likely explained by the different ancestry of the cohorts, cell types analyzed, and methodology. However, many of these studies either utilized unfractionated B cells or total naive B cells that included rN, aN, and transitional subsets, precluding an understanding of the regulation of the individual B cell populations (7, 8). Similar limitations apply to important studies across multiple autoimmune diseases using a genome-wide expression quantitative trait locus (eQTL) analysis, which identified a cluster in SLE determined by DN2 B cells (9). Interestingly, however, these studies demonstrated, at least in the Japanese population, that the expression of SLE risk variants was mostly enriched in disease-state signatures rather than disease-activity signatures (7).

Thus, to formally understand the contributions of disease state and disease activity to the abnormal regulatory programs of SLE B cells, we performed a direct comparison of the transcriptional and epigenetic landscape of carefully fractionated rN B cells and their earlier transitional precursors in active and inactive cohorts. Our results demonstrate that in SLE, rN B cells regardless of disease activity express an abnormal molecular program that is associated with disease state. These results extend the phenotype seen previously in rN from highly active individuals (5) and presumably reflect SLE intrinsic abnormalities. While sharing some of the interferon signatures of active disease rN cells, inactive disease rN cells displayed unique transcriptional enhancement of various cytokine and CD40 stimulation gene sets, suggesting the contribution of separate signaling pathways in priming SLE rN cells for subsequent activation and differentiation. In contrast, active disease SLE rN B cells displayed signatures that represented TLR- and/or IFN-driven stimulation conditions. Epigenetically, inactive rN cells exhibited differential chromatin accessibility modifications, particularly at IFN response gene loci. Finally, a distinct SLE transcriptomic signature was also present in the earliest peripheral precursors of naive B cells, namely, T1/T2 transitional B cells. Overall, these data suggest that SLE B cells harbor distinct alterations to their naive transcriptome and epigenome and that these alterations reflect both the disease state and activity.

## Results

### Resting and activated B cell subtypes are altered by SLE disease and disease activity.

The frequency of total naive B cells has been shown to inversely correlate with SLE disease activity in studies that did not exclude the aN fraction (2, 10). Additionally, there is a direct correlation between disease activity and increased numbers of aN cells as well as the DN2 progeny of aN cells (2). These B cell abnormalities were present in the individuals assessed herein as determined by multiparameter flow cytometry analysis of isolated peripheral blood PBMCs (Supplemental Figure 1A; supplemental material available online with this article; https://doi.org/10.1172/JCI199528DS1) from a cohort of 14 healthy individuals treated as controls (HCs), 46 with inactive SLE (SELENA-SLEDAI ≤ 4), and 22 with active SLE (SELENA-SLEDAI ≥ 5) (Supplemental Tables 1 and 2). On average, transitional B cells were increased (*P* = 0.0500) in both SLE cohorts as previously described (Supplemental Figure 1B) (11).

The frequency and total number of rN cells were decreased in active disease compared with HC subjects and negatively correlated with SLEDAI (Supplemental Figure 1, C and D). The frequency and number of rN cells were similar between inactive disease and HC. As expected, the aN population was expanded as a fraction of total CD19^+^ cells and as a fraction of total naive B cells in active SLE (Supplemental Figure 1E). Within the IgD^–^CD27^–^ DN compartment, we confirmed the expansion of DN2 cells (CD19^+^CD27^–^IgD^–^CD21^–^CD11c^+^) in active disease (2) and to a lesser but still significant degree in individuals with low-disease activity (Supplemental Figure 1F). A similar profile was observed for the DN3 cells (CD19^+^CD27^–^IgD^–^CD21^–^CD11c^–^), previously described in severe COVID-19 infection (12) and in IgG4-related disease (13). Combined, SLE cohorts displayed an increase of the DN2/DN1 and DN3/DN1 ratios, reflecting a reversal of the DN1 dominance within the DN B cell compartment characteristic of physiological B cell homeostasis (Supplemental Figure 1G). This effect was also seen in antibody-secreting cells (CD19^+^CD27^++^CD38^++^) (Supplemental Figure 1H). Reduced frequencies of unswitched memory B cells (CD19^+^CD27^+^IgD^–^) were observed in both SLE conditions (Supplemental Figure 1I), whereas switched memory B cells (SWM; CD19^+^CD27^+^IgD^+^) were unchanged by SLE diagnosis or disease activity (Supplemental Figure 1J). Overall, the B cell profiles of this SLE cohort align with our earlier findings (2, 3, 10).

Principal component analysis (PCA) using all gated B cell populations revealed that the samples from HCs were enriched in rN and unswitched memory B cells according to the principal component loadings, with considerable overlap with inactive SLE, while active individuals separated by PC1, reflecting their increased aN/DN2/antibody-secreting cell populations (Supplemental Figure 1K). The inactive SLE cohort was spread between SWM/DN1, T1/T2, and active disease–associated areas. Collectively, the B cell profiles of most inactive SLE showed more heterogeneity than those individuals with active disease. Importantly, inactive disease B cell profiles did not completely overlap with those from HC samples, a finding consistent with the hypothesis that B cells in inactive SLE may have unique molecular determinants.

### SLE rN B cells express an abnormal transcriptome in patients with low disease activity.

Our previous work demonstrated the presence of an SLE-specific transcriptome and epigenome in several B cell subsets of individuals with high disease activity, including rN (5, 6). To determine whether this transcriptome reflected a disease-determined regulatory program or was instead secondary to disease activity, RNA-seq was performed on cells isolated from PBMCs derived from an additional 17 HC, 15 inactive SLE (SELENA-SLEDAI ≤ 4), and 12 active SLE (SELENA-SLEDAI ≥ 5) individuals using a previously developed sorting strategy (14, 15) to rigorously sort rN B cells (CD19^+^CD27^–^IgD^+^MTG^–^CD24^+^CD38^+^) (5) and T1/T2 cells (CD19^+^CD27^–^IgD^+^MTG^+^CD24^+^CD38^+^) (Supplemental Table 1 and Supplemental Figure 2A). Here, the inactive disease cohort represented a longitudinal blend of individuals who were inactive and flare free from 1.6 to 107.3 months (Supplemental Figure 2B). PCA performed on the differentially expressed genes (DEGs) detected from pairwise comparisons of inactive SLE, active SLE, and HC, showed separation of each group of rN cells (Figure 1A). PC1 distinguished active SLE from both HC and inactive SLE and was positively correlated with SLEDAI (Figure 1B), whereas PC2 separated SLE samples from HC. Principal components were also analyzed with respect to other covariates, including age and medications, and revealed that these covariates were not correlated with the major PCs (Supplemental Figure 2, C and D). Thus, the data reflect that disease state and activity account for the dominant sources of variance in the data.

Differential expression analysis of rN B cells showed hundreds of DEGs between both disease activity groups and HCs and each other (Figure 1, C–E, Supplemental Figure 3A, and Supplemental Dataset 1). In general, both rN and T1/T2 B cells from individuals with active SLE displayed greater numbers of DEGs than their inactive SLE counterparts compared with HCs (Supplemental Figure 3A). PCA of T1/T2 cells also showed a similar separation, albeit with greater overlap between inactive and active SLE, as relatively few genes met FDR significance for differential expression between these 2 groups (Supplemental Figure 3, B and C). Thus, both rN and newly emerging T1/T2 B cells in inactive disease display hallmarks of an SLE disease state with a greater effect of disease activity on the transcriptome of rN cells. This pattern is consistent with the existence of a disease program initiated during early development, as reflected by T1/T2 B cells that expand during maturation into naive B cells and are modified during naive B cell activation. Additionally, many of the DEGs that are unique to either active or inactive SLE rN cells could dictate the properties of these cells in the different phases of the disease. For example, the upregulation in active disease of *TNFRSF13B* (TACI) (Supplemental Figure 3D), which binds BAFF and APRIL, could lead to alterations in tolerance (16). Upregulation of *FCRL5* in active disease (Supplemental Figure 3D) could inhibit anergy and facilitate TLR signaling, as suggested from mouse models (17). In contrast, inactive disease rN cells overexpressed *SPI1*, which encodes the ETS transcription factor (TF) PU.1, and the epigenetic regulator *EZH2* (Figure 1, D and E), a pattern consistent with preferential differentiation of inactive disease naive B cells through germinal centers (GCs) (18, 19).

### Type I IFN response, TLR, and BCR signaling genes differentiate rN B cells in active SLE.

A hallmark transcriptional feature of SLE is the upregulation of type I IFN response in multiple B cell populations (5, 20). Indeed, gene set variation analysis (GSVA) showed that the IFN-α response gene signature was present in the majority of individuals with SLE in this study (Figure 1F). Importantly, 2-way ANOVA of the GSVA results showed that the IFN-α response in rN cells was significantly different across HC and SLE samples but not between active and inactive disease.

A combined analysis of all cell types and activity levels highlighted limited DEG overlap between the groups (Figure 2A). The exceptions to this were primarily a core set of IFN response genes (*N* = 25) upregulated in all SLE groups (Figure 2B). In addition, 76 DEGs were shared only among the active disease rN and T1/T2 cells (Figure 2C). These genes were either upregulated or downregulated in active disease cells, while remaining at HC levels in inactive SLE. Of the genes upregulated (Figure 2D), *ISG15*, an IFN-induced antiviral response gene, has been associated with SLE disease activity (21). *NLRP6*, also overexpressed in active disease, encodes a key pattern recognition sensor component of inflammasome-mediated innate immunity and can be upregulated via type I IFN in some infections (22). The IFN regulatory factor 7 TF, encoded by *IRF7*, was more highly expressed in active disease B cells. IRF7 is a key regulator of the interferon response gene program (23) and genetic susceptibility to severe SLE (24). Of note, IRF7 mediates TLR7 signaling, a central SLE activation pathway (4). Finally, although *THEMIS2* expression was significantly enriched in active versus HC, some inactive disease rN and T1/T2 samples also expressed high levels of *THEMIS2* (Figure 2D). *THEMIS2* encodes a protein known to interact with PLCγ2 to lower B cell activation thresholds to low-avidity antigens, a function of particular relevance to naive B cells (25). *THEMIS2* is also of interest for SLE pathogenesis, as it is required to induce the expression of TBET and the production of IFN-γ (26), factors of major significance in the activation and differentiation of SLE naive B cells.

Downregulation was also observed for PI3K family members, including the catalytic subunits PIK3CD and PIK3CG, as well as the regulatory subunit PIK3R5 and the TF FOXO1, which regulates PIK3R5 (27) (Figure 2, C and E). The PI3K/FOXO1 axis is an important regulator of B cell activation and homing to the lymph nodes, and its attenuation may enhance class switch recombination in primary B cells (28). Other examples of modulators of lymphocyte activation gene sets downregulated in active disease included *FCRL3* and *FCER2* (encoding CD23), although there appears to be a downward trend in inactive disease (Figure 2E). FCRL3 is an inhibitor of BCR signaling, and stimulation through FCRL3 enhances TLR9-mediated B cell activation but suppresses further differentiation to plasma cells (29). CD23 is induced by stimulation of B cells with IL-4, while being reduced by IFN stimulation. Human CD23 expression is strongly downregulated in SLE aN B cells (3). Also downregulated in B cells within the active SLE cohort was the expression of *MEF2C* (Figure 2, C and E), a TF involved in B cell development and GC formation (30, 31).

### Inactive SLE has a unique transcriptional program.

To better understand the nature of a SLE disease-state signature in the absence of disease activity, all pairwise DEGs were hierarchically clustered resulting in 4 distinct gene clusters (Figure 3A). In parallel, the samples were K-means clustered into 3 groups based on gene expression patterns. This approach resulted in a clear group of high SLEDAI active SLE samples that also displayed the largest transcriptional distance from HCs. Although heterogeneity exists, the majority of inactive individuals formed a distinct cluster. Cluster 1 contained DEGs highly expressed in both SLE groups and was enriched for IFN response genes (Figure 3), many of which were shared between rN and T1/T2 cells (Figure 2B). This cluster also included the gene for STAT4, a TF induced by type I interferons that participates in IL-12 signaling, promoting IFN-γ production (32, 33). Polymorphisms in *STAT4* are associated with severe SLE (34), and its expression was elevated in rN cells from both disease activity levels, although to a greater extent in active SLE (Supplemental Figure 3D). Genes in cluster 2 were most highly expressed in active SLE, as exemplified by *THEMIS2*, also overexpressed in active transitional cells (Figure 2E). Cluster 2 also contained overexpressed members of the TNF superfamily, including *TNFRSF10A* (Death Receptor 4 or TRAIL receptor), which activates NF-κB inflammatory pathways. This cluster also contains the gene for STAG3, a protein critical for functionality of meiotic cohesin complexes, previously found as one of the top overexpressed genes in unfractionated CD19^+^ SLE B cells (35).

The remaining 2 clusters (3 and 4) contained genes that were down- and upregulated in inactive disease compared with HCs, respectively, and included gene sets broadly associated with cytokine responses, proliferation, and regulation of cell activation pathways (Figure 3, A and B). Cluster 3 comprised genes most severely repressed in active SLE and to a lesser extent in inactive SLE. Notably, it included *IL4R*, which counteracts the ability of IFN-γ to induce a TBET program in pathogenic SLE B cells as well as the differentiation of TLR7-activated naive B cells into pathogenic DN2 B cells (36–38). Cluster 3 also included downmodulated genes associated with B cell activation, naive B cell identity, and BCR signaling inhibition, including *PIK3R5*, *MEF2C*, and *FCRL3* discussed above (29, 30) as well as *NFAT1* (NFATC2), which is important for B cell anergy in a mouse self-tolerance model (39, 40). Cluster 4 contained genes highly expressed by inactive SLE rN B cells, including *BCL6*, which is required for the early stage of GC formation (Figure 3C) (41). Cluster 4 also contained genes associated with B cell activation and TNF-α/NF-κB signaling gene sets (Figure 3B), which suggests the potential for dysregulation of additional cytokine pathways in inactive SLE. To test this, we curated a library of gene sets representing various forms of B cell activation and cytokine signaling (Supplemental Table 3). In addition to enrichment of type I and II IFN gene sets in all SLE cell types and activity levels (Figure 3D), significant enrichment in inactive SLE rN B cells was found for CD40 signaling and several cytokine pathways, including TNF-α, IL-12, and IL-10, compared with HC and active SLE (Figure 3G and Supplemental Figure 3E). TNF-α/NF-κB and CD40 signaling pathways showed the greatest number of gene set members upregulated (FDR < 0.05) in inactive SLE (Figure 3E). It is important to note that many of these pathways have common overlapping genes and that all genes in a pathway may not be expressed in a given comparison.

We next interrogated the leading-edge subset of genes driving the association with CD40 signaling. That gene subset, with the greatest expression in inactive SLE, included members of not only the B cell CD40 response but also NF-κB signaling and GC B cell expressed genes (Figure 3F). Direct gene expression comparison between active and inactive SLE showed enrichment in inactive SLE for CD40 signaling, IL-4 response, and light zone GC B cell–expressed genes (Figure 3G). Compared with active disease, transcripts for GC B cell genes *BCL6* (Figure 3C) and *ITGB2* (Figure 3H) were significantly elevated in inactive SLE, suggesting that inactive SLE rN cells may be predisposed to interact with T cells and to follow distinct differentiation paths when activated. Alternatively, increased GC genes could restrict the differentiation potential of these B cells. Other significantly elevated transcripts in this comparison included *CD44*, a cell adhesion molecule with maximal expression in preactivated naive B cells and the TF *BHLHE40*, which is often associated with B cell activation and may restrict GC formation (Figure 3H) (42, 43). Increased expression of *TNIP2*, which encodes a modulator of NF-κB signaling, was observed in inactive SLE compared with HC (Figure 3H). Intriguingly, reduced expression of *TRAF5* (a signaling mediator of CD40 signaling) (44, 45) in inactive SLE compared with HC suggested that this activation pathway may be dysfunctional to some extent (Supplemental Figure 3D). Overall, these data suggest an increased role of abnormal CD40, NF-κB, IL-4, and TNF-α cytokine signaling events in inactive disease rN B cells.

### Epigenetic alterations to IFN-α response and TLR regulation loci remain accessible during disease remission.

To characterize the epigenetic state of rN B cells during active and inactive SLE, we performed the assay for transposase-accessible chromatin with sequencing (ATAC-seq) (46) on a subset of the same SLE and HC samples that were profiled by RNA-seq above (Supplemental Table 1). A total of 24,793 differentially accessible regions (DARs) were found after comparing both disease groups and HCs in all pairwise combinations (Supplemental Dataset 2). Similar to the RNA-seq data, correlation analysis of the major principal components to other covariates revealed PC1 correlated with disease activity and SLEDAI and not to medications or other covariates (Supplemental Figure 2D). Both inactive (Figure 4A) and active SLE (Figure 4B) exhibited thousands of DARs (FDR < 0.05) when compared with HCs. On average, DARs present only in inactive disease exhibited small changes in accessibility, whereas those that overlapped both disease groups and active disease had higher absolute differential accessibility levels (Figure 4C). When assessing a 2-fold change, one-third of DARs from active disease rN cells overlapped with those occurring in inactive rN cells (Figure 4D). This collection of loci reflecting inactive and active disease-specific DARs, as well as those that overlapped, was used for gene pathway enrichment analysis (Figure 4E).

This analysis showed significant enrichment for overlapping and nonoverlapping pathways in each disease group. A subset of regions was significantly changed in accessibility only in inactive SLE. Consistent with the reduced effect size for this set of DARs, subtle increases in accessibility in DARs within the *NOTCH3*, *ETS1*, and *DGKD* loci were observed for inactive SLE rN cells (Supplemental Figure 4A). *NOTCH3* and *ETS1* encode transcriptional regulators, and *DGKD* encodes a diacylglycerol kinase. DARs specifically associated with active SLE were enriched around genes in several signaling cascades and in the positive regulation of lymphocyte activation, including *SOCS1*, which encodes a suppressor of IFN signaling (47) and a direct regulator of IRF7 degradation (48). DARs at *CLEC16A*, which functions in autophagy and intracellular trafficking, were enriched in active SLE (Supplemental Figure 4B). *CLEC16A* is associated with susceptibility to autoimmune disease and inflammation (49, 50) and is located at an autoimmunity risk locus (51) that includes the MHC-II master regulators *CIITA* and *SOCS1*. The *HKDC1* and *SUPV3L1* genes, which encode a hexokinase and a mitochondrial double-stranded RNA helicase, respectively, contain DARs found only in active SLE (Supplemental Figure 4B).

Importantly, DARs in both disease activities (when compared with HCs) included regions with increased accessibility around several IFN response genes, such as *IFI44/IFI44L* and *RSAD2* (Figure 4F and Supplemental Figure 4C). To determine whether these changes to chromatin accessibility were correlated with gene expression changes in SLE, expression was queried for those genes found at loci with at least 1 shared DAR in both active and inactive SLE. Many of these genes showed continued differential expression from HCs in both disease activity groups; however, a significant increase in the magnitude of expression changes was observed in active SLE individuals (Figure 4G). These data suggest that inactive disease rN B cells maintain accessible regions of chromatin around important loci, such as type I IFN response genes, indicating that either durable epigenetic memory, persistent inflammatory signaling, or both is occurring. The increase in overall gene expression at these shared loci suggests that additional events/factors are present/augmented in the active disease state.

### Unique and shared TF motifs in inactive and active SLE epigenomes.

To determine which TFs may play a role in regulating genes with differential accessibility in inactive and active SLE, enrichment analysis of known TF-binding motifs was performed on all DARs. Ranking the magnitude of enrichment of motifs over genomically matched background regions using HOMER (52) identified specific TF families that were enriched in each of the above categories of DARs. TF binding motifs for EBF1, p63, and TCF4, which contribute to mature B cell identity, homeostasis, and GC formation (53–56), were enriched in DARs with increased accessibility in inactive disease (Figure 5A). DARs in both disease activity groups (but not HCs) were enriched for ETS and IRF family motifs. Regions that became less accessible only in active SLE were enriched for NF-κB, FOXO1, and SPIB motifs, whereas inactive-specific DARs with less accessibility were enriched for EGR family motifs (Figure 5B). EGR motifs were previously shown to drive a network of genes in rN cells of active SLE (5). The enrichment for IRF family motifs, including IFN-stimulated response elements, was found within regions that remained accessible in both disease activity levels. However, additional enrichment of IRF and IFN-stimulated response element motifs was also found in DARs that were accessible only in active SLE (Figure 5C). Basic helix-loop-helix (bHLH) family motifs, such as those for TCF4, E2A (TCF3), and n-MYC, were also enriched with the highest ranking in the DARs specific to inactive SLE (Figure 5D). Intriguingly, significant enrichment of IRF8 motifs was found in regions both losing and gaining accessibility compared with HCs, particularly in active disease DARs, but also in those shared with inactive disease (Figure 5E). As above, NF-κB motifs were only enriched in DARs that lose accessibility in SLE (Down DAR), most significantly in active disease rN (Figure 5E). Together, these data suggest roles for ETS, IRF, bHLH, EGR, and NF-κB TF families in establishing or maintaining SLE disease activity–associated epigenetic changes.

### MEF2C and E2A/E2-2 factors likely regulate inactive SLE-specific gene sets.

RNA expression and chromatin accessibility assay data were integrated into a gene regulatory network with the Taiji tool (57), which uses the PageRank algorithm to reveal the factors most implicated in coordinated regulation of RNA expression based on chromatin accessibility (Figure 6A). Similar to previous results (5), ATF3 was enriched in both active and inactive rN datasets, suggesting a role in driving the common SLE signature. *PAX5* and *RUNX1* had higher PageRank values in inactive disease for both rN and T1/T2 cells, while *EGR1* and *IKZF1* values were highest for the T1/T2 cell type within inactive SLE. *TCF4*, *TCF3*, and *TCF12*, which encode the TFs E2-2, E2A, and HEB, respectively, along with *MEF2C* and *MEF2D* showed the highest PageRank score in rN cells from inactive disease samples. In contrast, the factors NFATC2 and NFATC3 showed reduced importance in inactive disease rN cells compared with healthy or active counterparts (Figure 6A). This reduced importance correlated with lowered expression of the NFAT genes themselves (Figure 6B). A comparison of PageRank scores between active versus inactive disease showed that TFs such as TCF3, MEF2D, and CEBPB were predicted to be more important in inactive disease rN cells. Other PageRank scores were likely less driven by changes in their own transcript expression, but more by their influence on target genes. For example, *BCL6* was more highly expressed in inactive disease but appears to have a lower impact on its target genes, as assessed by PageRank.

To test this, TF gene targets in inactive rN cells, as determined by accessibility of motifs at predicted enhancer regions, were cross-referenced across the B cell activation and cytokine-signaling gene set libraries used in Figure 3. Fisher’s exact tests showed significant enrichments of target genes of PageRank-implicated TFs (Figure 6C). For example, IRF3 and IRF7 regulatory targets were most significantly enriched for IFN response genes, supporting their known role in TLR-mediated type I IFN response gene expression pathways (58). Targets of MEF2C/D and NR3C2, as well as to a lesser extent RUNX1 and NF-κB factors, were enriched for elements of the CD40 signaling gene set, which was expressed more highly by inactive rN B cells. *MEF2C* expression was significantly decreased from HCs in active SLE only (Supplemental Figure 3D). NFATC1 and NFATC2 target genes were enriched for B cell receptor and TLR signaling genes, suggesting alterations in the potential for these cells to respond to future antigen, cytokine, or stimulation challenges. The regulatory targets of E2A (*TCF3*), HEB (*TCF12*), PRDM1, and the glucocorticoid receptor NR3C1 were enriched for a gene set consisting of the DEGs from active versus inactive SLE in rN cells (Figure 6C). Thus, these data connect epigenetic changes in DARs with their associated TF motifs and alterations in gene expression pathways regulated in inactive SLE.

## Discussion

Serological, genetic, genomic, and functional analyses identified abnormal B cell regulation as a major pathogenic component of SLE. The centrality of this abnormality is also supported by the discovery of monogenic defects that operate in a B cell–intrinsic fashion (4) and the striking therapeutic improvement that follows the profound B cell depletion induced by CD19 CAR T cells (59). Thus, understanding the nature of B cell dysregulation in SLE is of great importance to unravel disease pathogenesis. Physiologically, analysis of naive B cells provides a window into the programming of preantigenic, resting B cells, which determines the intensity and quality of their response to different stimulatory conditions. We had previously shown that in Black individuals with active SLE, circulating B cells exhibit alterations in their cellular phenotypes and epigenomes, including the sharing of TF-driven regulomes by several peripheral B cell populations (5). Of note, these alterations of normal B cell physiology were present in naive B cells conventionally considered to be resting (antigen-naive) (60). This finding led us to hypothesize that SLE naive B cells are abnormally regulated by a disease-associated program. Given that our original study concentrated on high disease activity individuals, we could not exclude the possibility that disease activity rather than disease state might be responsible for the changes observed.

Here, we demonstrate that a distinct SLE disease phenotype consisting of transcriptome and epigenome changes is harbored by rN B cells of individuals with SLE of primarily Black ancestry, irrespective of disease activity. Importantly, we also identified additional changes that are associated with higher disease activity, prominently including the differential transcriptional regulation of TLR, BCR, costimulatory (CD40), and/or cytokine-driven activation pathways, as well as epigenetically regulated gene programs, in particular as it pertains to IFN-induced programs. Combined, our data are consistent with a model where the SLE disease state would prime rN B cells for abnormal responses to self-antigen stimulation and toward skewed effector B cell differentiation through either extrafollicular or GC pathways, likely contributing to the determination of B cell endotypes, as recently shown in our studies of mRNA vaccine responses in SLE (43).

Although not as pronounced, we found that the newly emerging T1/T2 B cell populations also expressed a unique transcriptional signature that is distinct from HC subjects. Transitional B cell frequencies here and previously reported (11), were found to be increased in patients with SLE, and their development to mature cells includes an important peripheral tolerance checkpoint that is dysregulated (61). Our results confirm that SLE T1/T2 cells exhibit a persistent IFN response gene program irrespective of disease activity and including IRF7 overexpression, a feature consistent with the initiation of TLR7 hyperresponsiveness in T1 cells in lupus mouse models (11, 62). However, many of the transcriptional and epigenetic aspects that differentiate rN cells in individuals with active or inactive disease were not found in the immature T1/T2 cell type, possibly reflecting the fact that these newly emerging cells have not been sufficiently exposed to the factors that drive the inactive SLE program in rN cells.

Despite stringent sorting of rN cells, both inactive and active SLE B cells showed transcriptomic and/or epigenetic evidence of abnormal activation pathways not seen in healthy individuals. In active SLE, this signature is reminiscent of that shared across other circulating B cell populations (5). For example, the type I IFN response has previously been highlighted as a part of the transcriptional signatures associated with SLE across many cell types in the blood (20). Genes responding to IFN are upregulated in both inactive and active disease, and the epigenetic changes associated with expression of many of these genes remain dysregulated in the B cells of individuals during symptom remission. Importantly, however, a small portion of IFN genes were specific to active disease. These results suggest that it is possible that IFN signaling in B cells is a constant feature of SLE in Black populations and only partially correlated with onset of symptomatic disease. Alternatively, previous disease flares/episodes may have resulted in the imprinting of an epigenetic memory of the IFN response that persists in the remaining B cell compartment during inactive disease. A combination of both scenarios may also occur. It is plausible that such epigenetic alterations in turn lead to a continued disease gene expression program and may even predispose individuals to future flare events. Evidence of cellular memory through chromatin remodeling after IFN stimulation has been previously documented (63). Many such regions enriched for IFN response genes and TLR signaling are found to be more accessible in both active and inactive SLE rN cells; however, the relatively lower expression of these genes in the inactive state suggests that other factors, such as a potential increase in an acute inflammatory environment associated with active SLE or loss of additional tolerance control mechanisms, may be required for manifesting a fully active IFN disease signature.

Curiously, some DARs appear in both disease states for genes that are neither differentially expressed nor expressed in the RNA-seq data sets but nonetheless seem to have relevance to disease. For example, DARs for both the *RSAD2* and *SOCS1* genes were identified. *RSAD2* encodes the IFN-induced and SLE-associated gene Viperin, which has been shown to promote TLR7 and TLR9 signaling in dendritic cells (64, 65) and is overexpressed in Sjögren’s syndrome (66). These data add additional evidence for a baseline role of TLR7 in early activation of SLE B cells, a feature consistent with the pathogenic contribution of this pathway in a B cell–intrinsic manner (4, 67). Knockdown of the gene *RSAD2* led to attenuated B cell hyperactivity, cell viability, and NF-κB signaling in those cells. *RSAD2* is not detectably expressed in the rN B cells in this study, suggesting that the open chromatin state may reflect an ability for this gene to respond more quickly to stimulation or to be induced as the cells differentiate further after receiving additional disease-associated activation signals. Several DARs in the *SOCS1* gene were more accessible in active disease, yet *SOCS1* is also not a DEG in the rN populations studied. *SOCS1* is induced by TLR7 stimulation and IFN (68) and negatively regulates cytokine signaling, including IRF7 and IFN responses (48). IRF7 was found to be less important in controlling its target genes in T1/T2 cells from individuals with active SLE, suggesting that there is still some control of the IFN response in these emerging cells and further supporting how longer-acting environmental changes may primarily affect rN cells in active disease.

We determined that inactive SLE rN cells also exhibit a unique and abnormal activation phenotype, with altered expression of genes involved in several cytokine signaling or activation response pathways. Inactive SLE rN cells showed increased expression of TNF-α, IL-4, IL-10, IL-12, and CD40/CD40L signaling pathway genes compared with active SLE and in some cases HC rN cells. It is important to point out that there is often overlap in genes in many of the signaling pathways, which typically include genes/proteins that are necessary for a signaling event to occur within a cell. For example, the IL-12 signaling pathway includes TYK2 and JAK2, which are also important for IFN signaling. Regardless of the overlap, these data suggest that some aspect of the inactive SLE environment may involve rN B cells that maintain relatively higher potential to receive these signals compared with active disease. Changes in IL-12 signaling could potentially sensitize cells to receive IFN-γ signals and enhance B cell differentiation pathways (69). IL-4 stimulation of rN B cells inhibited DN2 B cell formation under IFN conditions and represents an important regulator for maintaining a quiescent rN state by promoting unique differentiation pathways distinct from those produced by IFN or TLR signaling (37). It is plausible that these activation control pathways may be functional only in inactive disease, as exemplified by the reduced expression of some IFN response or activation-modulating genes (e.g., *ISG15*, *THEMIS2*). Additionally, in inactive rN cells, *TRAF5*, an important mediator of both TLR and CD40 signaling, continued to be downregulated in a manner reminiscent of SLE DN2 cells (2). Increased CD40L signaling pathways in inactive SLE rN cells is consistent with the negative effect of sustained signaling on naive B cell differentiation (2). This suggests that CD40 signaling may be initiated or persistent in inactive disease and decreases during the active phase of the disease, a model consistent with both the enhanced contribution of a B cell extrafollicular pathway and the decreased CD40L signaling of the DN2 cells generated through that pathway (2, 3).

The issue of whether the transcriptome and epigenetic alterations observed in B cells in the context of inactive SLE disease is intrinsic or a result of extrinsic factors is manifold. From the extrinsic side, there was clearly a response to cytokines such as IFN, IL-4, IL-12, and TNF that are manifested in both the gene expression patterns and chromatin accessibility. From the intrinsic side were changes in BCR receptor signaling pathways (PIK3 genes), GC-related transcripts (*BCL6*), and CD40 signaling. While these pathways, in particular the BCR and CD40 pathways, contain disease risk alleles and are more likely to intrinsically influence B cells, they both could also be extrinsically influenced by external factors such as the levels and immunogenic properties of self-antigens. Moreover, changes in the epigenetic landscape may result in future changes in gene expression as disease activity increases. Thus, it is likely that both intrinsic and extrinsic factors converge to control SLE disease.

Here, we found that B cells in inactive SLE have extensive and complex changes to accessible chromatin at regions of importance for SLE pathogenesis, including IFN response loci. The presence of these alterations suggests that at least some part of the epigenetic programming associated with active disease represents a memory of previous events or has occurred due to persistent exposure to disease mediators, such as low levels of inflammation or cellular signaling. Such stimulation would not be sufficient to fully activate or differentiate the B cells. The enrichment of IRF family TF motifs at these regions implicates these factors in controlling the long-term effect of disease on the epigenome. IRF TFs play a central role in B cell development at many stages (70) and have been shown to modulate TLR signaling (71). Although direct biochemical data would be required to designate specific factors with epigenetic and transcriptional changes, the ability to designate TF binding motifs is a first step in understanding transcriptional networks driving SLE. Some TFs, such as the EGR family factors, which have been implicated in the active SLE signature (5), appear to be more specific to active disease, whereas E2A, E2-2, and MEF2C were associated with B cells found in the inactive disease state. E2A and E2-2 have been shown to cooperatively regulate GC B cell and long-lived plasma cell development in mice (72). The accessible chromatin enrichment of these motifs in inactive rN B cell chromatin suggests that binding of these factors may be occurring or that these regions are readily accessible for future activation signals and represent a predisposition toward GC B cell programming. This is also supported by increased expression of GC B cell–associated genes in both inactive and HC individuals, which is suppressed in the active SLE rN B cell phenotype. Active disease also showed the lowest expression of the TF MEF2C. Loss of MEF2C has been found to lower T-dependent responses in B cells as well as GC formation (30). Overall, these data suggest that E2A/E2-2 and MEF2C may play a role in driving the unique programming found in inactive SLE. The loss of these programs in active disease may shift differentiation pathways for the rN population away from GC and toward extrafollicular development, partially explaining the increase in these populations in active disease.

In summary, we show the separate effects of disease state and disease activity in SLE rN B cells in a group of primarily Black individuals with SLE. Importantly, in both disease activities, the rN B cell population is distinct from those of healthy individuals and displays transcriptional and epigenetic signatures that indicate that the SLE environment influences their future activation and differentiation potential. The fact that the newly emerging T1/T2 B cells also display an altered signature supports a role for the SLE environment on influencing these cells. Differences in TLR, CD40, IL-4, and IFN pathways in active and inactive SLE denote molecular differences in disease state but also suggest their potential to dictate the future differentiation path that these cells take as disease progresses. Given these cells’ potential for differentiation into many of the cell types associated with pathogenicity in SLE, future studies considering the unique molecular programming of both active and inactive rN cells can provide insight into important new treatments for minimizing, predicting, or preventing future SLE flare events.

## Methods

### Sex as a biological variable.

Sample details for all cohorts are in Supplemental Table 1. Data were collected from predominantly Black female individuals, who represent an understudied population with a high incidence of SLE. Clinical information was collected from electronic medical records. All individuals diagnosed with SLE met the 2019 EULAR/ACR criteria according to their medical records. Disease activity was evaluated with SELENA-SLEDAI scores by rheumatologists. Individuals with a score of 5 or greater were defined as active and those with a score less than or equal to 4 as inactive.

### Flow cytometry analysis and sorting cells.

Whole blood was drawn from each subject by venous puncture using blood collection tubes with sodium heparin (Becton Dickinson). To isolate PBMCs, whole blood was poured into LeucoSep (Greiner) or SepMate (STEMCELL Technologies) tubes with Ficoll with the same volume of PBS and centrifuged at 1,000*g* for 10 min at room temperature. Isolated PBMCs were washed with 50 mL PBS, and red blood cells were removed using ACK Lysing Buffer (Thermo Fisher Scientific). Three million PBMCs were stained with the antibody mixture for B cell profile screening after Fc receptor blocking. Antibodies used in this study are detailed in Supplemental Table 2. The cells were fixed by 0.8% paraformaldehyde and analyzed on an Aurora (Cytek) flow cytometer.

For cell sorting, thawed frozen cells were washed with prewarmed complete media (RPMI-1640, 10% FBS, 1% l-glutamine, and 1% HEPES) and centrifuged at 500*g* for 10 min. The cells were resuspended and incubated with 5 mL of media containing 20 nM of MitoTracker Green FM, Invitrogen/Thermo Fisher Scientific (catalog M7514). for 30 min at 37°C in 5% CO_2_ and then centrifuged at 500*g* for 5 min. To release the excess MTG, cells were resuspended and incubated with 5 mL prewarmed complete media for 30 min at 37°C in 5% CO_2_. PBMCs were centrifuged at 500*g* for 10 min at room temperature and resuspended in PBS with 0.5% BSA, 5% normal rat serum, and 5% normal mouse serum. Cells were incubated with the antibody mixture for sorting for 30 min at 4°C. Cells were washed twice with 5 mL PBS, centrifuged at 500*g* for 5 min, resuspended, and incubated with 100 μL PBS with 0.1 μL Fixable Viability Dye eFluor 506 (Thermo Fisher Scientific) for 15 min at 4°C. Stained cells were washed with PBS containing 0.2% FBS and resuspended using sorting buffer (PBS with 2% FBS, 1% EDTA, and 2.5% HEPES). rN (CD19^+^, CD27^–^, IgD^+^, MTG^–^, CD24^mid^, and CD38^mid^) and T1/T2 B cells (CD19^+^, CD27^–^, IgD^+^, MTG^+^, CD24^+^, and CD38^+^) were sorted on a FACS ARIA II (BD Biosciences). Sorted cells were collected in 300 μL RLT buffer for RNA-seq and 300 μL RPMI-1640 with 10% FBS for ATAC-seq. The same patients and cell sorts were used for rN and T1/T2 RNA-seq and ATAC-seq.

### RNA-seq.

RNA from cells sorted for sequencing was isolated using the QuickRNA Microprep kit (Zymo Research, R1050). Subsequently, the SMART-Seq v4 Ultra Low Input RNA Kit (Takara Bio, 634894) was employed for cDNA synthesis, with 400 pg of cDNA utilized as input for the NexteraXT kit (Illumina) to generate sequence libraries. The DNA libraries were then sequenced at the Heflin Center for Genomic Sciences (University of Alabama at Birmingham, Birmingham, Alabama, USA) using a NextSeq 500 platform (Illumina).

### RNA-seq data analysis.

The raw sequencing data were aligned to the hg38 reference genome using STAR v.2.5.3 (73). Duplicate reads were removed using Picard (Broad Institute; http://broadinstitute.github.io/picard/). Quality control was performed on all samples, and those with total uniquely mapped read quantities less than 5 × 10^6^ were excluded. Additional samples were removed after PCA indicated them as outliers. For the T1/T2 cell types, 1 sample of each SLE group was excluded, leading to 14 inactive and 11 active samples. Gene expression counts were normalized as reads per kilobase per million (RPKM). This normalization was achieved by analyzing the coverage across all exons specific to unique ENTREZ genes, with the assistance of the GenomicRanges package (74). All further analysis was performed in R. Differential expression testing was conducted using DESeq2 (75). To exclude effects of confounding variables on DEG discovery, technical batch and self-reported race were used in the glm model for DESeq2. Transcripts were determined to be differentially expressed if the FDR was < 0.05 and absolute log_2_ fold change (log_2_FC) between conditions was ≥ 0.5. GSVA was conducted using the GSVA package (76). PCA was performed on RPKM values (scaled by gene) using the princomp function from the stats package in R (v3.6.2; R Core Team; https://www.R-project.org/). Covariate correlation analysis was performed by calculating a coefficient of determination for each covariate versus each principal component. GSEA (77) was conducted using a list of all detected genes ranked by multiplying the sign of the fold change by –log_10_ of the *P* value. The ComplexHeatmap R package (78) was used for generating heatmaps and for hierarchical and K-means clustering utilizing the hclust function from the stats package. Information on the source of the curated gene sets used can be found in Supplemental Table 3.

### ATAC-seq.

Isolated cells from each sample were prepared for ATAC-seq as previously described (79). Briefly, cells were resuspended in a mixture containing 12.5 μL of 2× tagmentation DNA buffer, 2.5 μL of Tn5, 2.5 μL of 1% Tween 20, 2.5 μL of 0.2% digitonin, and 5 μL of H_2_O. The mixture was then incubated at 37°C for 1 hour. Subsequently, cell lysis was achieved by adding 2 μL of 10 mg/mL proteinase K and 23 μL of tagmentation cleanup buffer (326 mM NaCl, 109 mM EDTA, and 0.63% SDS), followed by a 30 min incubation at 40°C. The tagmented DNA was purified, and small fragments were selected using AMPure XP beads (Beckman Coulter, A63881). PCR amplification with dual indexing primers (Illumina, FC-131-2004) was performed to generate a sequencing library. The final libraries were purified and size-selected again using AMPure XP beads, their quantities were measured using QuBit (Life Technologies, Q33231), and size distributions were determined using a bioanalyzer (Agilent 2100). The libraries were pooled at equimolar ratios and sequenced at the Emory National Biomedical Research Center Genomics Core on an Illumina NovaSeq6000 using a paired-end 100 cycle run.

### ATAC-seq data analysis.

The raw sequencing data were aligned to the hg38 genome using Bowtie v1.1.1 (80). The PICARD tool was used to mark duplicates. Peaks of accessibility enrichment were identified using MACS2 v2.1.0 (81), and these peaks were then annotated to the nearest gene using HOMER (52). For quality control, samples with low numbers of uniquely mapped reads (<5 × 10^6^) and/or low fragments of reads in peaks (FRiP < 5%) were excluded, resulting in fewer ATAC-seq samples available for the final analyses. Fixed-width peaks sized at 501 bp in length were discovered and used for later quantitative analysis using the ChrAccR package (82) and a score per million cutoff of ≥ 5. Additionally, to be considered a viable peak, each must be discovered in at least 2 samples per analysis group. Coverage was determined for all of the reads at each peak region using the summarizeOverlaps function from the GenomicAlignments package and normalized as reads per peak million. DARs were determined using DESeq2 as peak regions that met the criteria of FDR ≤ 0.05 and absolute log_2_FC ≥ 0.5. To control for confounding variables during differential testing, surrogate variable analysis (83) was performed and the first 2 surrogate variables, as well as technical batch, were included as covariates in the glm model for DESeq2. Pathway enrichment for DARs was performed using GREAT (84). HOMER was used for known and de novo motif enrichment using the findMotifsGenome.pl function.

### Integrated analysis.

The ATAC-seq and RNA-seq data for each sample were integrated to create a gene regulatory network model using the Taiji program (57), which assigns the connections between TFs and their regulated genes based on expression and accessibility of motifs around gene locus. Personalized PageRank scores for measuring network centrality and the importance of TFs to the overall network were calculated using Taiji as previously described (85). To intersect the regulons of each TF with the gene sets previously described, overlaps between the TF target lists and gene sets were quantified and tested for enrichment using a Fisher’s exact test. The resulting *P* values were corrected for false discovery rate using the Benjamini-Hochberg method (p.adjust function in R).

### Statistics.

All statistical analyses were performed using R or GraphPad Prism (v10.0.2). Inference on flow cytometry data consisted of Tukey’s multiple-comparison test with 1-way ANOVA, Pearson’s correlation, or 2-tailed Student’s *t* tests as indicated. A *P* value < 0.05 was considered significant. Inference on RNA-seq or ATAC-seq count data was performed using DESeq2 to account for unequal variances across transcripts.

### Study approval.

Sample collection was approved by the Emory University IRB. Written informed consent was provided by all subjects.

### Data availability.

Sequencing data have been deposited in the NCBI Gene Expression Omnibus under accession numbers GSE262846 for RNA-seq and GSE263716 for ATAC-seq. Flow cytometry data are available upon request. Code and data processing scripts for the sequencing data are available at https://github.com/cdschar/Rose_SLE_diseaseActivity; commit ID 8bf0941. Values for all data points in graphs can be found in the Supporting Data Values file.

## Author contributions

JRR performed all informatics analyses and prepared the initial draft; as such, he was assigned the first of the co–first authors. YI performed experiments and analyzed data with JRR. SH generated multiomics libraries. SAJ performed experiments and designed sorting strategies. AK participated in the design of the clinical cohorts and collection of samples. CDS designed informatics pipelines with JRR and analyzed data. JMB and IS conceptualized the project, designed experiments, interpreted data, and completed the manuscript. All authors contributed to editing the manuscript.

## Conflict of interest

The authors have declared that no conflict of interest exists.

## Funding support

This work is the result of NIH funding, in whole or in part, and is subject to the NIH Public Access Policy. Through acceptance of this federal funding, the NIH has been given a right to make the work publicly available in PubMed Central.

- NIH U19AI110483 to JMB and IS and T32GM0008490 to JRR.
- Emory Integrated Genomics Core (RRID:SCR_023529).

## Supplementary Material

Supplemental data

Supplemental data set 1

Supplemental data set 2

Supplemental tables 1-3

Supporting data values

## Figures and Tables

**Figure 1:**
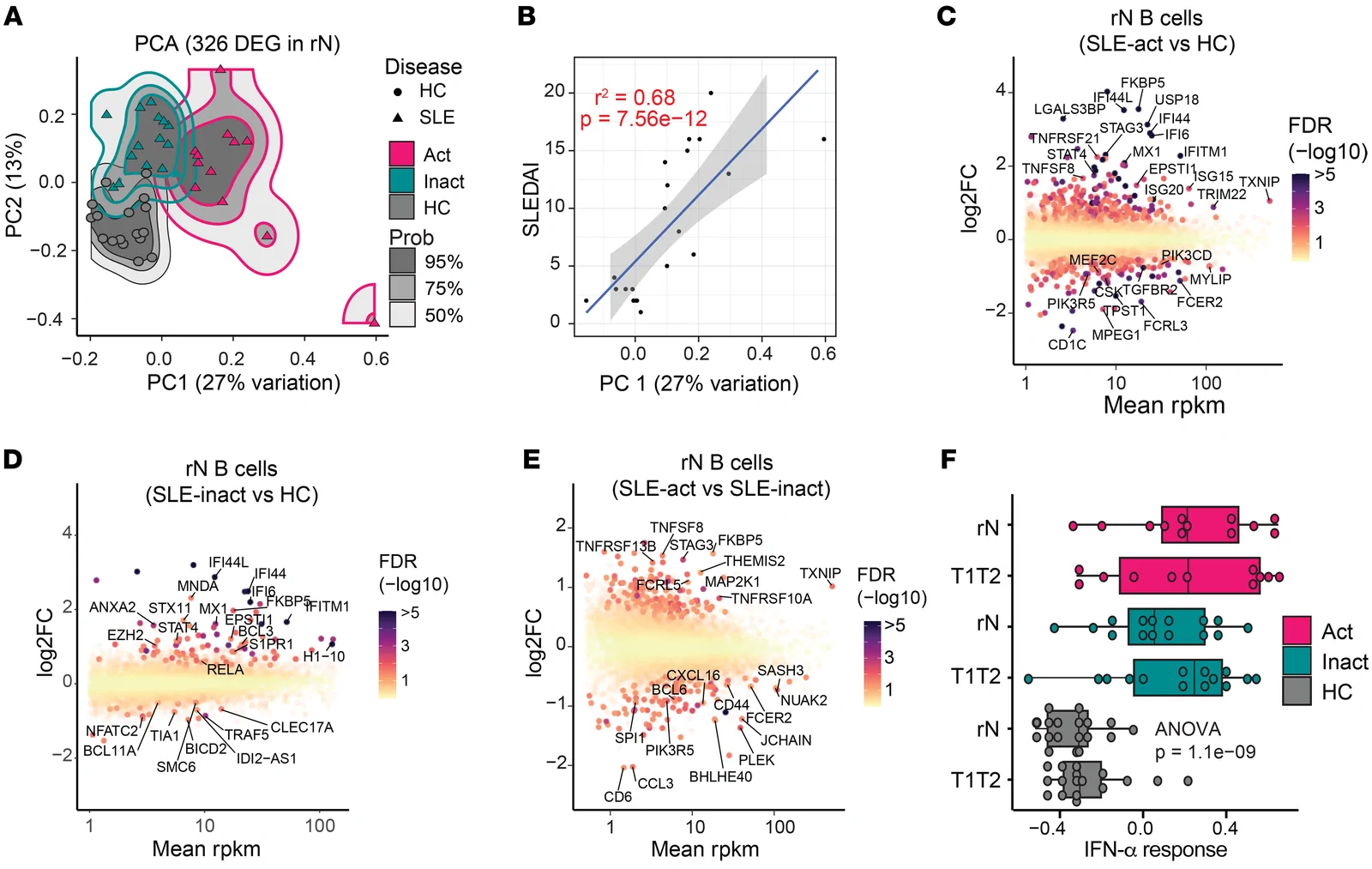
The distinct transcriptomes of active and inactive SLE B cells. (**A**) PCA plot of DEGs in rN B cells. Contours represent high-density regions calculated at probabilities (Prob). (**B**) Scatterplot of PC1 score by SLEDAI for SLE samples. *P* value and correlation were calculated by Pearson’s correlation test. (**C**–**E**). MA plots showing mean normalized expression (RPKM) for each gene by log_2_FC. Colors represent –log_10_ of *P* value derived from differential testing. (**F**) GSVA plot showing IFN-α response gene set. *P* values were calculated using 2-way ANOVA. Post hoc tests were performed across samples within the same cell type using Tukey’s honestly significant difference test and showed significance (adjusted *P* value < 0.05) in SLE versus HC comparisons but not between active and inactive SLE groups.

**Figure 2:**
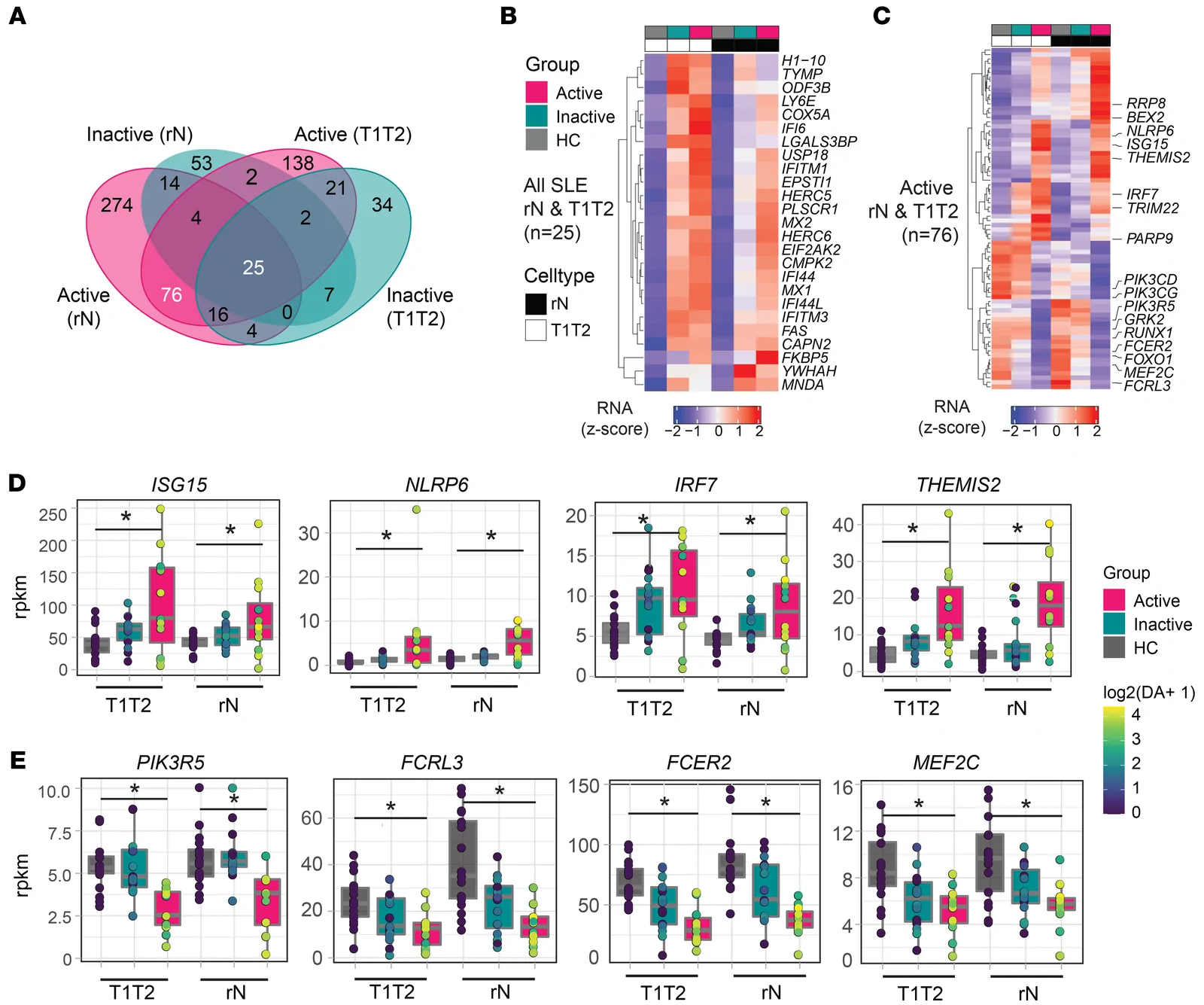
Shared and distinct gene expression signatures in active and inactive SLE. (**A**) Venn diagram showing the number of DEGs shared and distinct across groups. (**B**) Heatmap of the 25 DEGs shared across all 4 groups from **A**. Normalized expression values (RPKM) were averaged by group and *z* scored. (**C**) Heatmap as in **B** showing the 76 DEGs overlapping between the indicated cell types. (**D** and **E**) Box-and-whisker plots showing normalized gene expression for indicated genes and groups with higher or lower expression, respectively, in active SLE. Dots represent individual samples and are colored according to log transformation of SLEDAI score. The box-and-whisker plots depict the minimum and maximum values (whiskers), the upper and lower quartiles, and the median.

**Figure 3:**
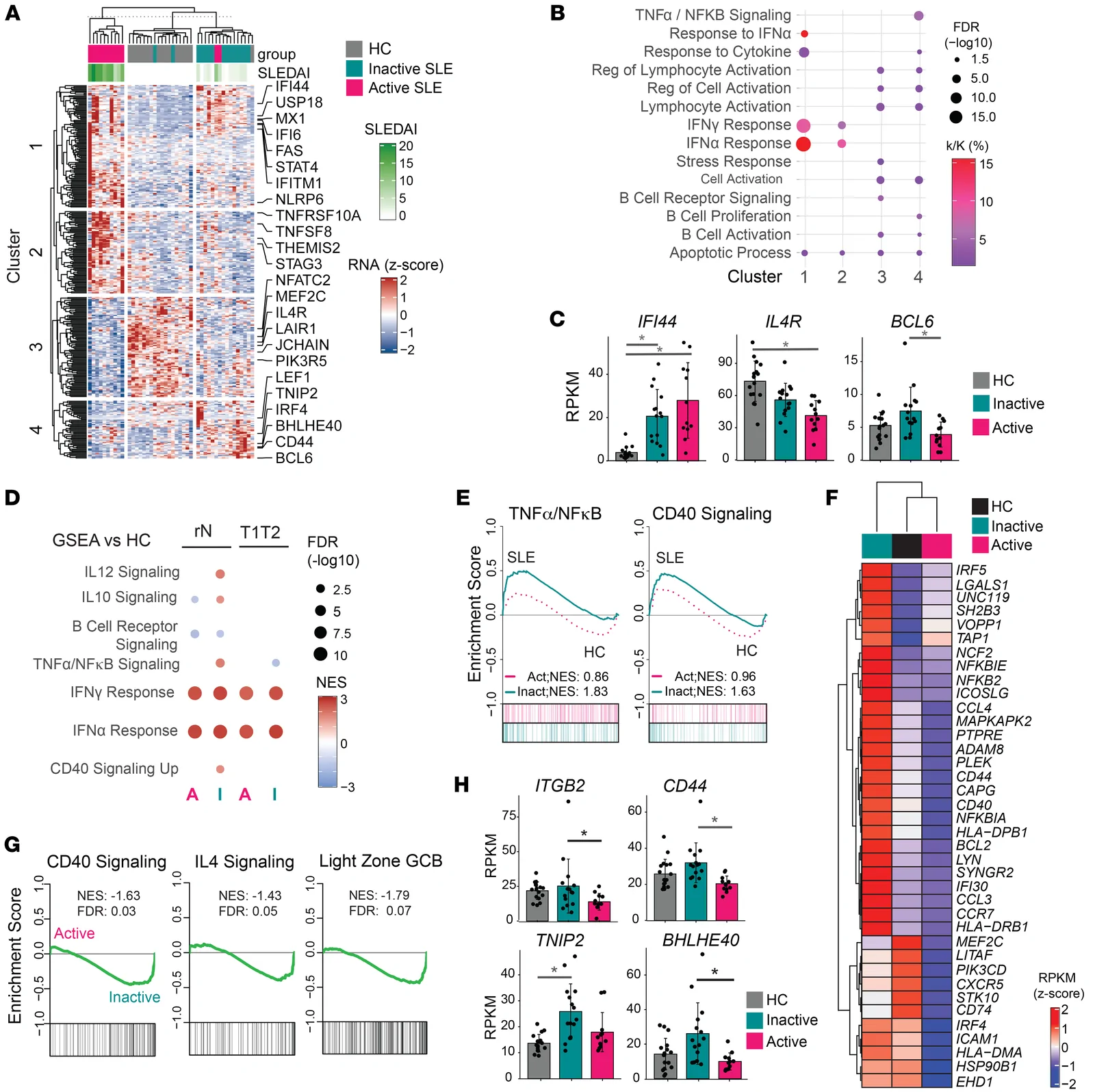
Cytokine and CD40 signaling gene expression in rN inactive SLE. (**A**) Heatmap showing DEGs in rN B cells with respect to disease activity. Normalized expression (RPKM) of DEGs were *z* scored and clustered using hierarchical clustering into 4 clusters by row, whereas columns were K-means clustered using a K = 3. (**B**) Bubble plot showing Gene Ontology enrichment of clusters from **A**. Bubbles are colored by the overlapped (k) genes normalized by each gene set size (K) and are presented as percentages. (**C**) Bar plots of normalized gene expression (RPKM) for indicated genes in rN cells. (**D**) Bubble plot showing GSEA. Bubbles are colored by normalized enrichment score (NES) and sized according to enrichment *P* value. Gene set descriptions are provided in Supplemental Table 3. A, active SLE; I, inactive SLE. (**E**) GSEA plots and NES for inactive or active SLE versus HC rN B cells. Dotted lines indicated enrichment that did not meet significance (FDR < 0.05). (**F**) Heatmap showing leading-edge genes from the CD40 signaling GSEA gene set in **E**. Normalized expression (RPKM) of DEGs were *z* scored. (**G**) GSEA plots for indicated gene sets for rN cells. (**H**) Bar plots of normalized gene expression (RPKM) for indicated genes from rN cells. In **C** and **H**, data are presented as the mean ±1 SD. Asterisks indicate significance in DESeq2 differential expression testing (Wald test with Benjamini-Hochberg correction), FDR < 0.05.

**Figure 4:**
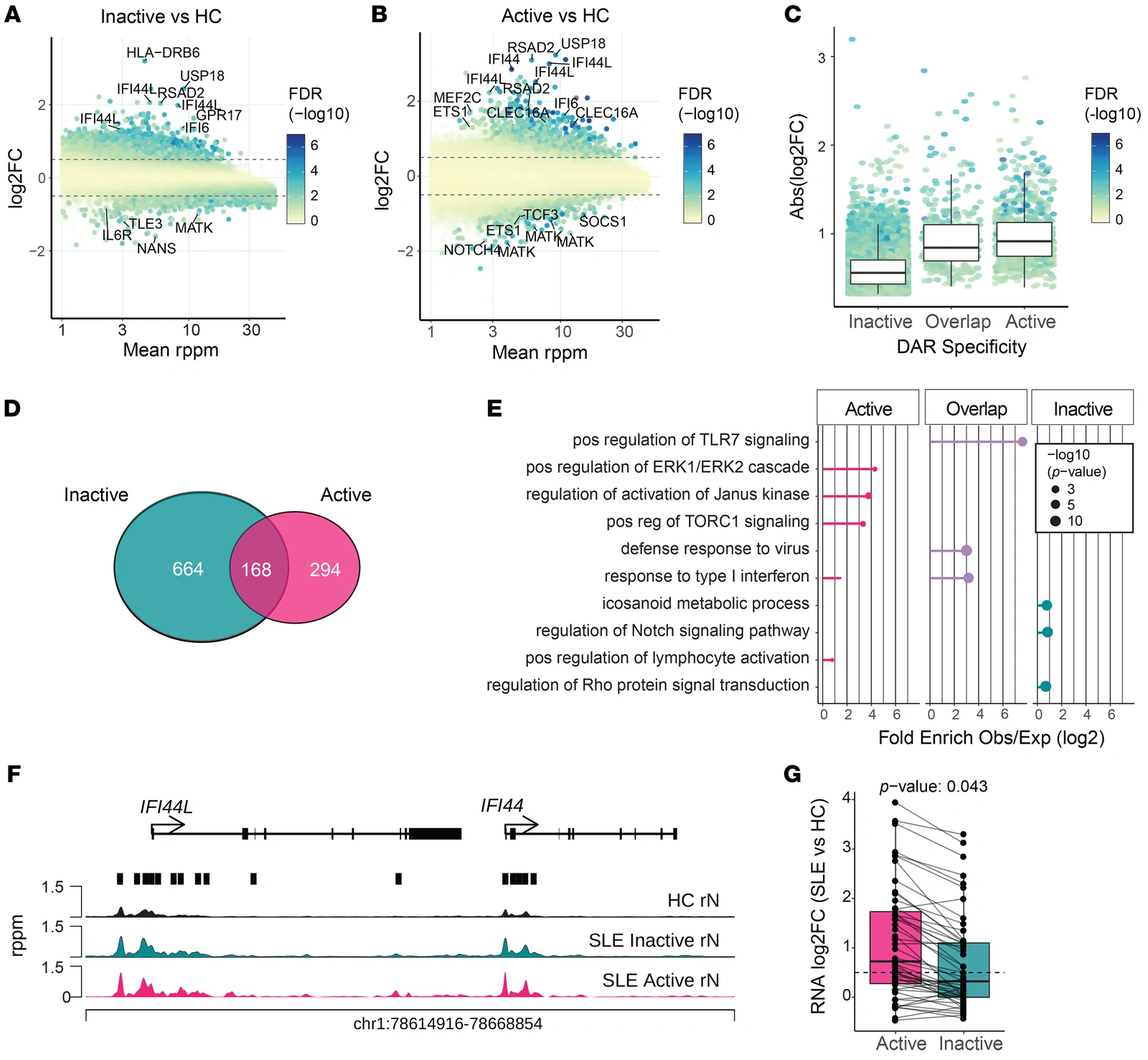
Chromatin accessibility changes in inactive SLE partially overlap those in active SLE. (**A** and **B**) MA plots showing mean normalized accessibility (reads per peak million [rppm]) for each locus by log_2_FC compared between the indicated groups. Dotted lines represent ± 0.5 log_2_FC cutoff. (**C**) Whisker-and-box plot showing absolute value of log_2_FC for loci in **A** and **B** separated by whether they occur in the indicated groups or overlap. (**D**) Venn diagram showing number of DARs in either disease activity compared with HCs using a log_2_FC cutoff of > 1. (**E**) Lollipop chart showing gene pathway enrichment of DARs (log_2_ of number observed [Obs]/number expected [Exp]) in the indicated groups. (**F**) Genome track plots. Black boxes represent DARs. (**G**) Whisker-and-box plots showing log_2_FC in gene expression in rN for each SLE group compared with HCs that have ≥ 1 shared accessible DAR. Lines connect individual genes. The box-and-whisker plots depict the minimum and maximum values (whiskers), the upper and lower quartiles, and the median. *P* value was calculated by paired 2-tailed Student’s *t* test.

**Figure 5:**
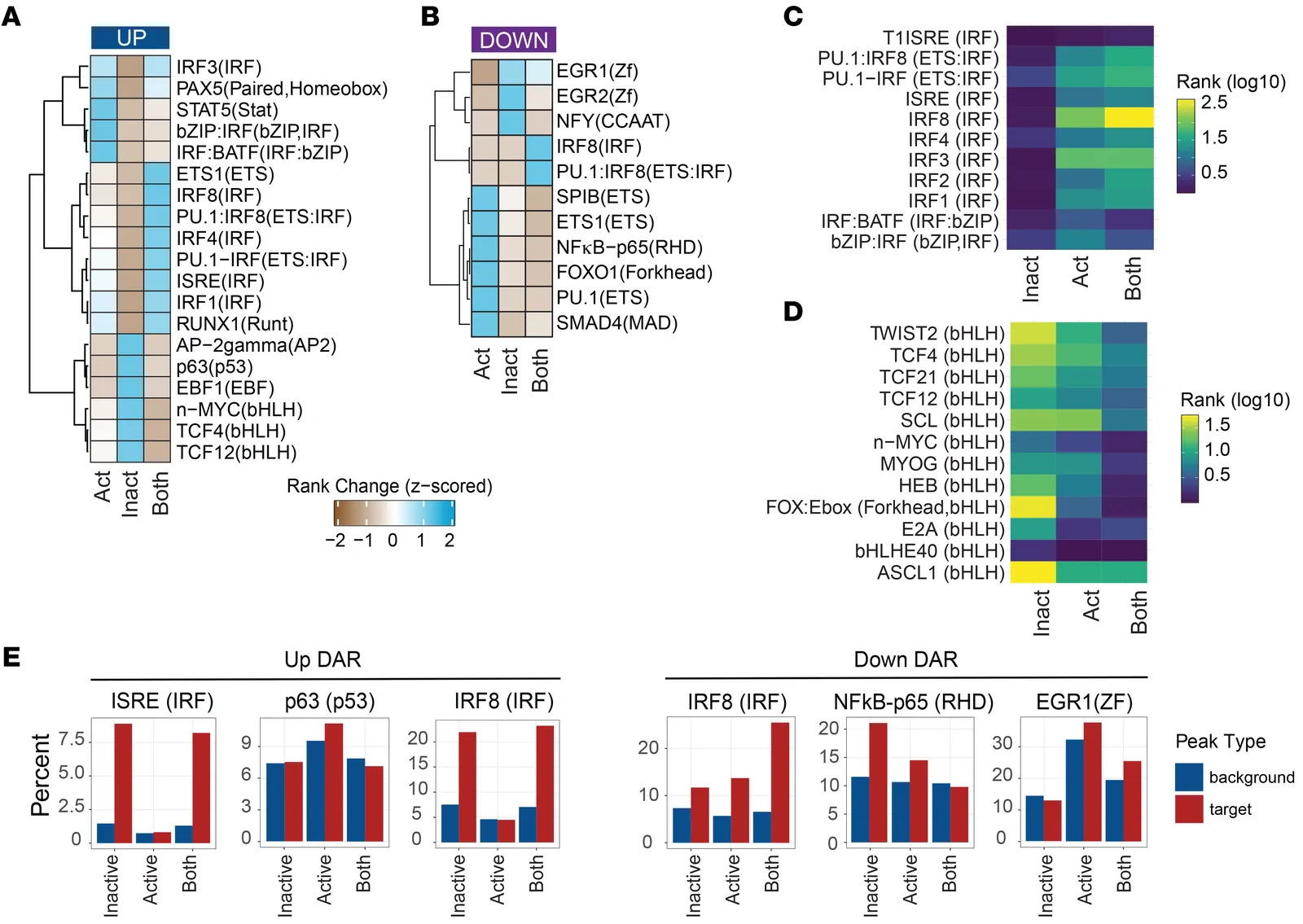
IRF and NFAT family factor motifs are enriched in shared elements of inactive and active SLE chromatin. (**A** and **B**) Heatmaps showing *z* scored ranking of HOMER motif enrichment from DARs with positive (**A**) or negative (**B**) log_2_FC compared with HCs ranked by effect size within each column. Columns represent specificity to active, inactive, or overlapped (those found in both disease states) DARs. (**C** and **D**) Heatmap of ranking for IRF (**C**) or bHLH (**D**) family factors after log_10_ transformation. (**E**) Bar plots showing percentage of regions containing indicated target motif (red) versus selected genomic background (blue). Act, active SLE; Inact, inactive SLE.

**Figure 6:**
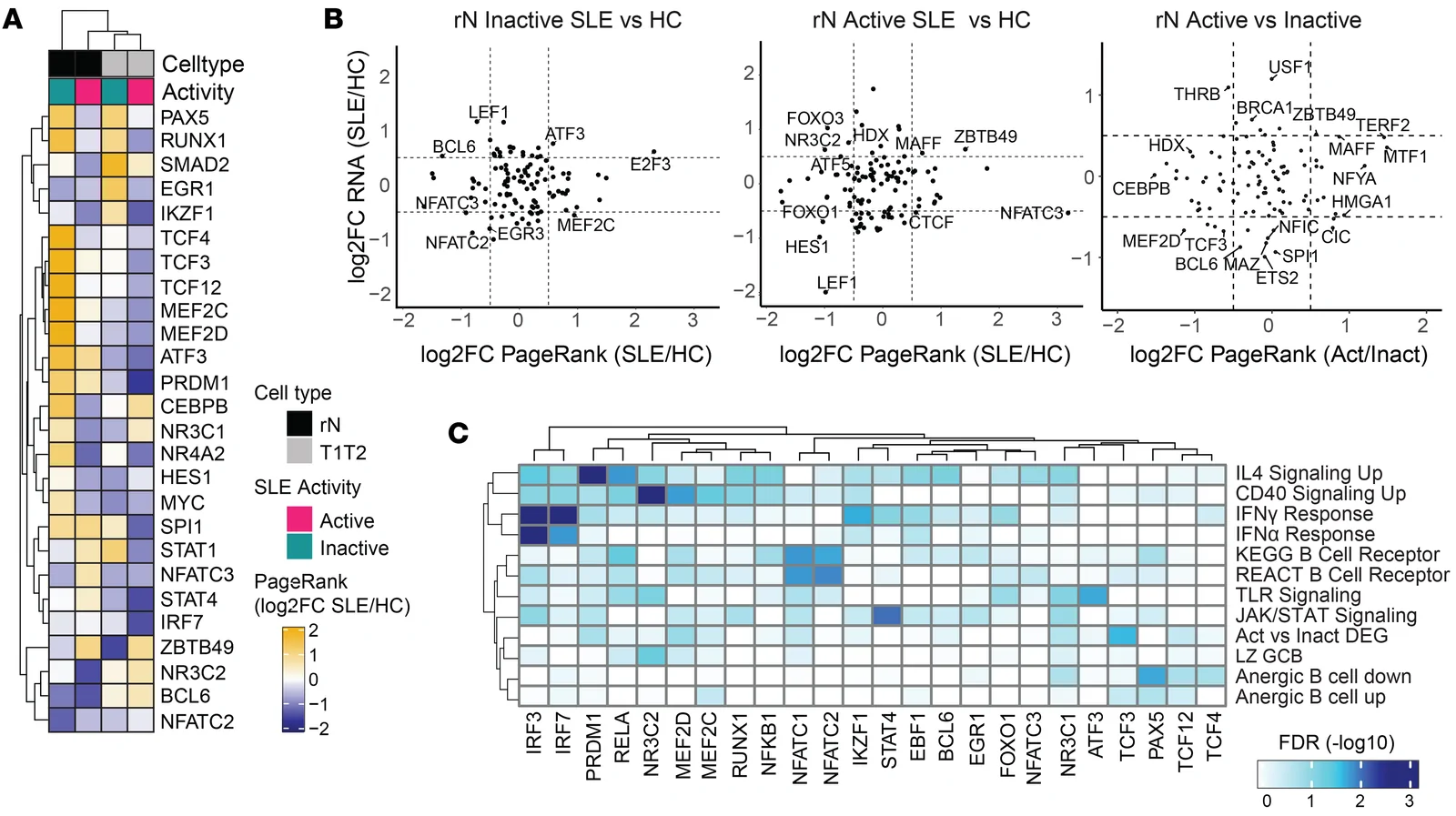
MEF2 and E2A/E2-2 regulate inactive SLE gene expression programs. (**A**) Heatmap showing log_2_FC of PageRank network importance score for SLE conditions (rN and T1/T2) versus HCs. (**B**) Scatterplots showing PageRank score (*x* axis) versus RNA expression. (**C**) Heatmap representing significance of overlap between TF target list and relevant B cell gene pathways. Color represents FDR *P* value of Fisher’s exact tests transformed using –log_10_.

## Version 1 10/15/2026

Electronic publication
