# Supplementary material for: Identification of a systemic lupus erythematosus disease signature in resting naive B cells
Source: J Clin Invest. 2026 Oct 15;136(20):e199528. doi: 10.1172/JCI199528 (PMC13668619; doi:10.1172/JCI199528)
Supplement: Supplementary file 1 [file jci-136-199528-s275.pdf]

Supplemental Figure 1

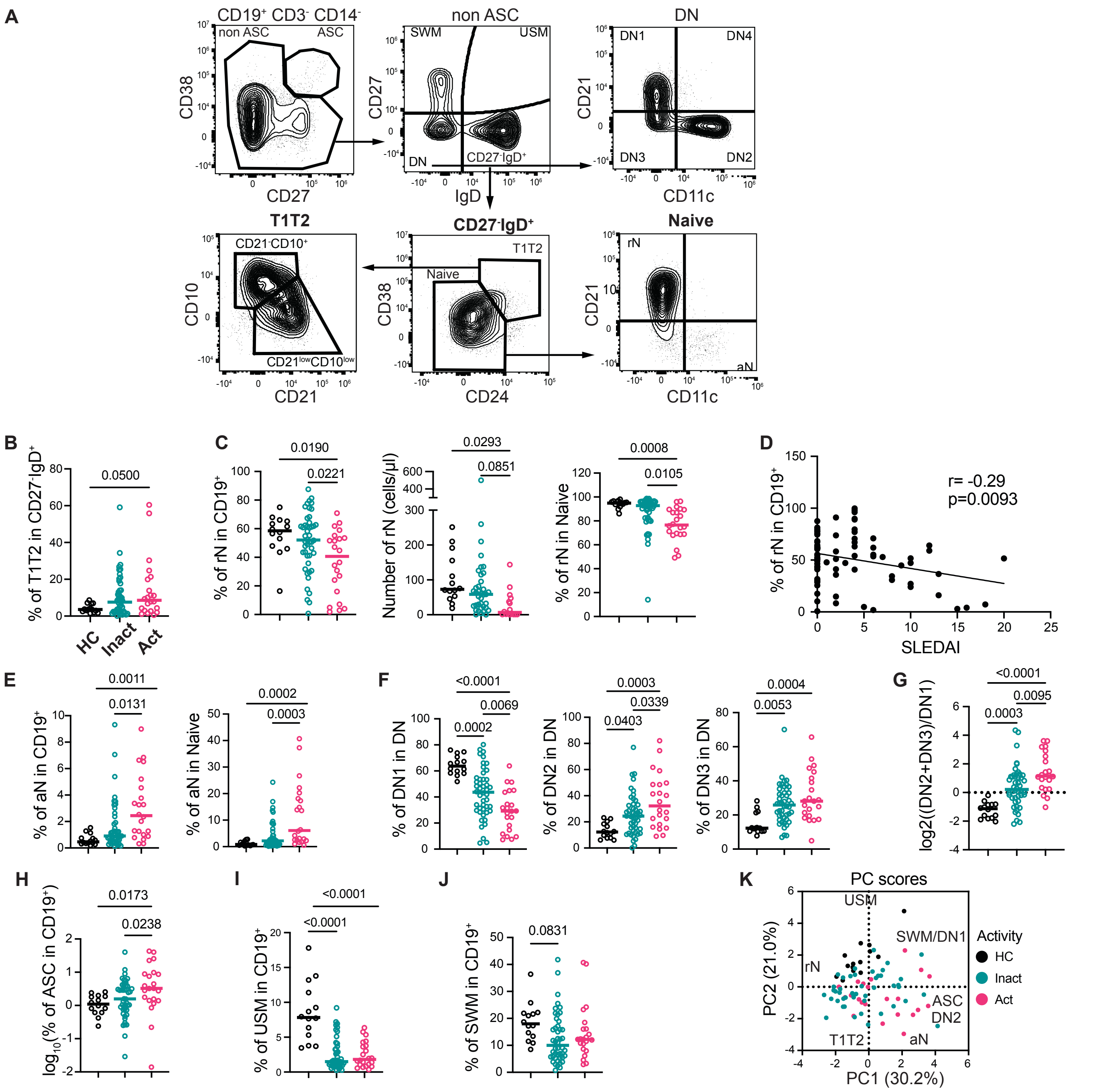

Supplemental Figure 1. Multiparameter flow cytometry analyses of SLE B cells across disease activities. A. The overall gating strategy for flow cytometric analysis is shown. B. The percentage of T1/T2 in CD27–IgD+ B cells. C. The percentage of rN in Naïve B cells. D. Percent of rN B cells plotted versus SLEDAI score. E. The percentages of activated naïve (aN) B in CD19+ B cells and in the naïve compartment. F. The percentage of DN1, DN2 and DN3 in DN. G. Ratio of DN2 and DN3 versus DN1 cells. H-J. The percentages of ASC, unswiched, and switched memory B cell (USM, SWM) in CD19+ B cells. K. Principal component analysis of flow cytometry results with the percentage of B cell population in CD19+ B cells in healthy control (HC), inactive SLE and active SLE. For panel D, the p-value and correlation were calculated by Pearson's correlation test. For all others, the p-value was calculated by Tukey's multiple comparisons test with one-way ANOVA. Bars indicate comparison for p-values < 0.1. Circles represent each individual and median bar is shown.

Supplemental Figure 2

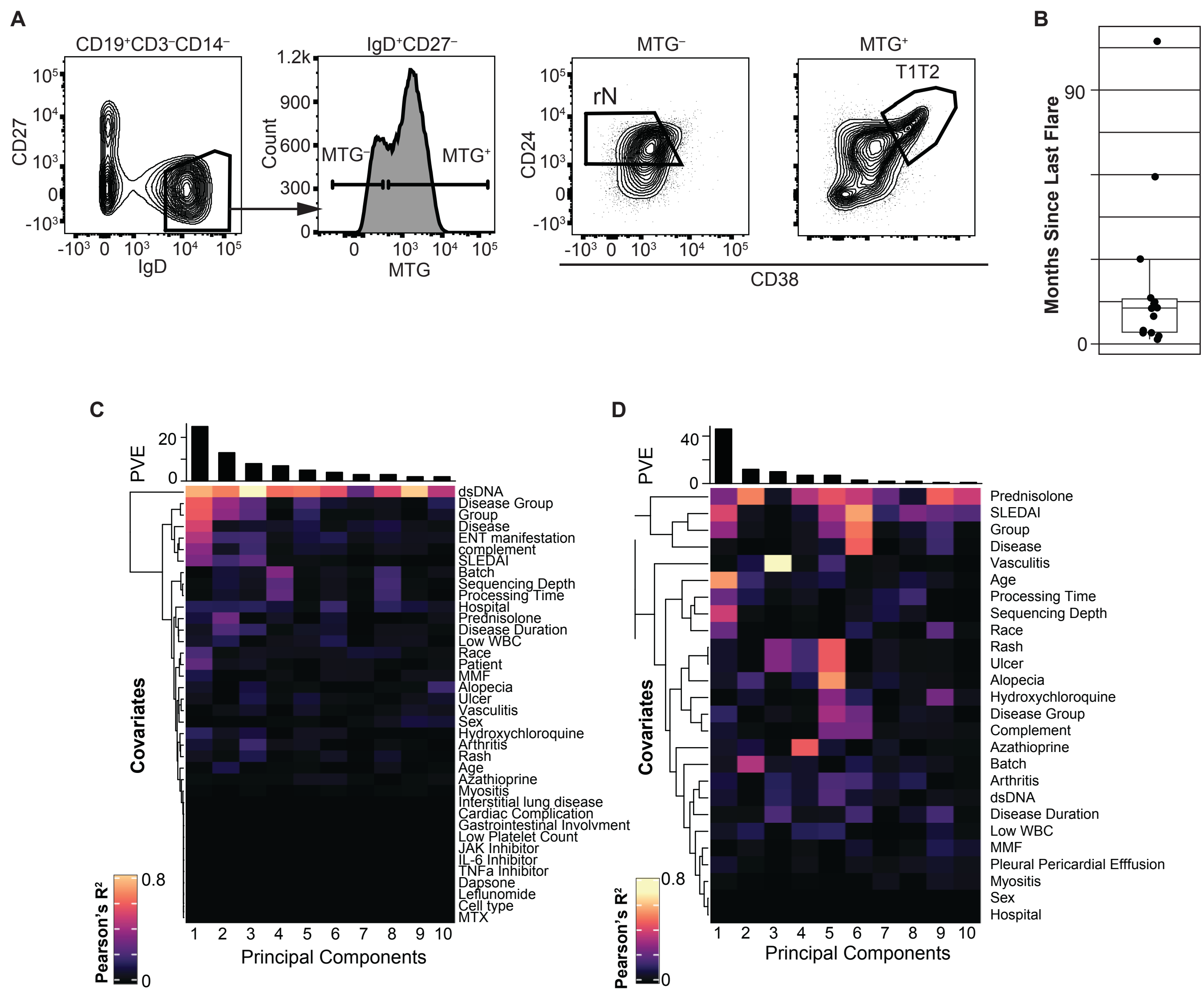

Supplemental Figure 2.
A. The gating strategy for FACS and sequencing analysis.
B. Plot of the time from last flare that inactive SLE subjects samples were collected.
C. and D. Covariate analysis of RNA and ATAC-seq data sets with respect to the principal components

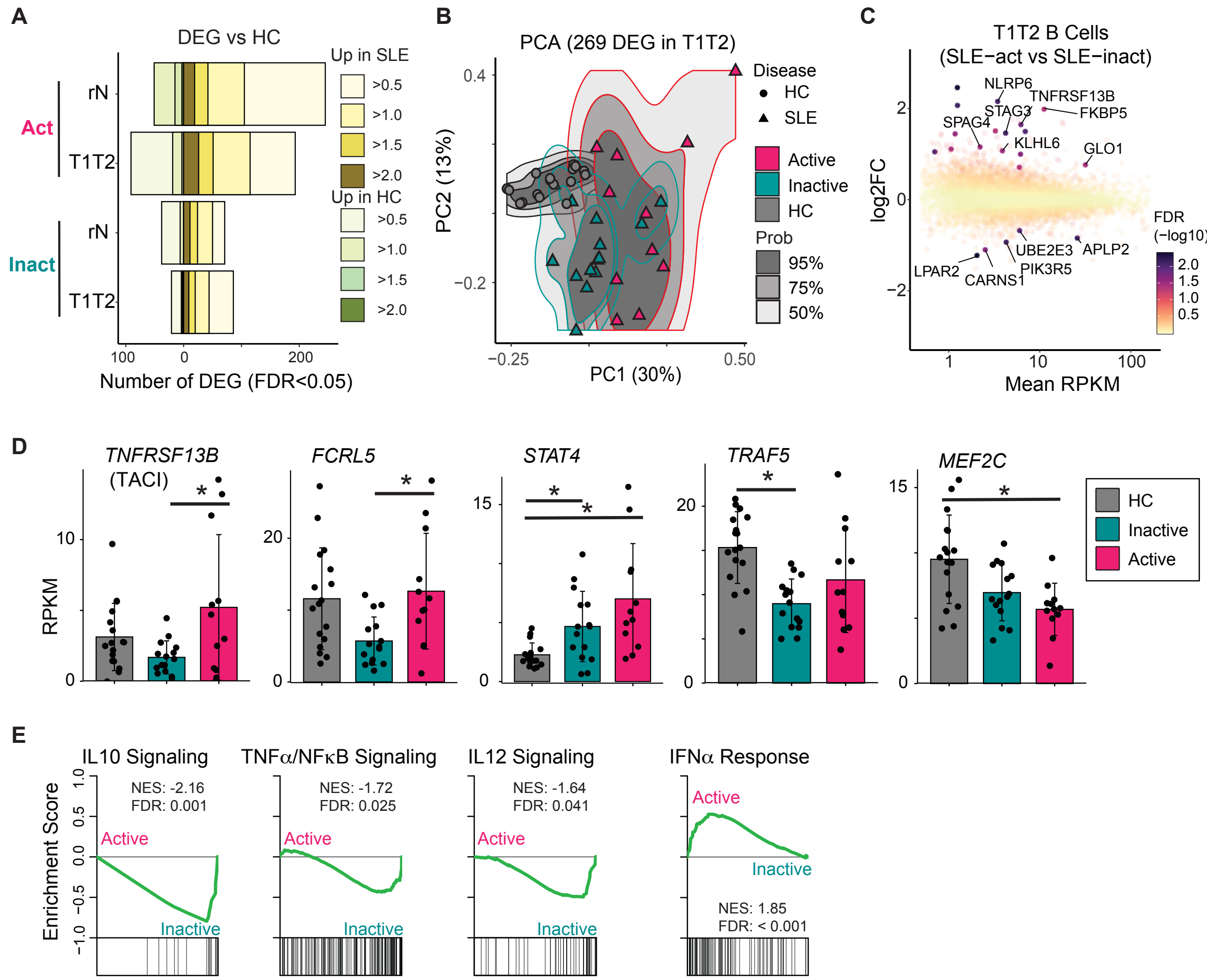

Supplemental Figure 3

A. Bar plots indicating the number of FDR significant DEG in each comparison in reference to HC group. Color represents log2 fold change. B. PCA plot comprised of 269 FDR significant DEG in T1/T2 cell type. Contours represent high density regions calculated at probabilities indicated by shading. C. MA plot showing average expression and log2FC between active and inactive T1/T2 cells in SLE. Colors represent -log10 of p-value derived from differential testing. Colors represent bins showing magnitude of log2FC values in either direction. D. Bar and dot plots of normalized gene expression (rpkm) in rN for indicated genes. Dots represent individual samples. Error bars represent ± 1 SD. Horizontal bars and asterisks represent significance in differential expression testing for indicated groups. E. GSEA plots for indicated gene sets showing enrichment, FDR, and normalized enrichment (NES) scores for active vs inactive SLE rN.

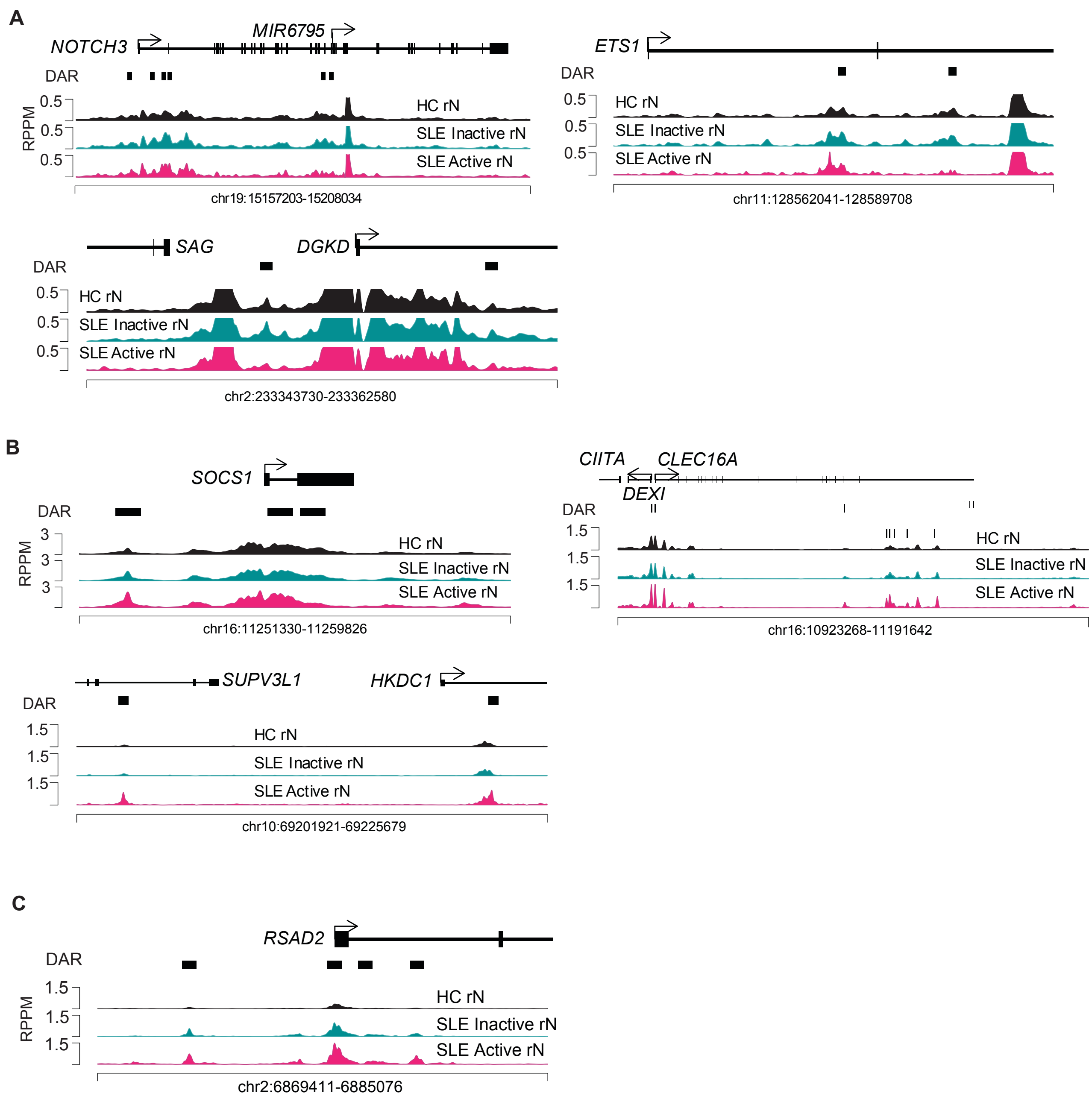

Supplemental Figure 4. Genome track plots showing normalized chromatin accessibility data (RPPM) around indicated loci with DAR specific to (**A**) inactive (**B**) active (**C**) active and inactive SLE. DAR are indicated by horizontal bars.
